# Anti-nociceptive and anti-inflammatory effects of hydroalcoholic extract and essential oil of *Pinus eldarica* in animal models

**DOI:** 10.22038/AJP.2021.18038

**Published:** 2021

**Authors:** Valiollah Hajhashemi, Behzad Zolfaghari, Pooya Amin

**Affiliations:** 1 *Department of Pharmacology and Isfahan Pharmaceutical Sciences Research Center, School of Pharmacy and Pharmaceutical Sciences, Isfahan University of Medical Sciences, Isfahan, Iran*; 2 *Department of Pharmacognosy, School of Pharmacy and Pharmaceutical Sciences, Isfahan University of Medical Sciences, Isfahan, Iran *

**Keywords:** Analgesic, Anti-inflammation, Herbal medicine, Pinus eldarica

## Abstract

**Objective:**

Several species of *Pinus* have shown anti-nociceptive and anti-inflammatory properties. This study was aimed to evaluate anti-nociceptive and anti-inflammatory effects of hydroalcoholic extract of bark and essential oil of leaves of *P. eldarica* in animal models.

**Materials and Methods:**

Hydroalcoholic extract of bark and essential oil of leaves of *P. eldarica* were prepared and phenolic content and essential oil composition were analyzed using Folin-Ciocalteu method and GC/MS, respectively. Anti-nociceptive effect was assessed using acetic acid, formalin and hot plate tests in male Swiss mice (25-30 g) and for evaluation of anti-inflammatory activity, carrageenan test in male Wistar rats (180-200 g) and croton oil-induced ear edema in male mice, were used. Involvement of opioid, α2-adrenergic, 5-HT3 receptors and adenosine triphosphate (ATP)-dependent K+ channels in pain relief was tested using naloxone, ondansetron, yohimbine and glibenclamide.

**Results:**

The total phenolic content of the extract in terms of gallic acid equivalent was 404.9±7.7 mg/g of the extract powder. *P. eldarica *hydroalcoholic extract (200 and 400 mg/kg) and essential oil (100 and 200 µl/kg) significantly (all, p<0.001) decreased pain behavior in acetic acid and formalin tests but not in hot plate test. The extract and essential oil suppressed edema in carrageenan and croton tests. Glibenclamide partially reversed the anti-nociceptive effect of hydroalcoholic extract while the other antagonists were ineffective.

**Conclusion:**

Hydroalcoholic extract of bark and essential oil of leaves of *P. eldarica* significantly decreased acute and chronic pain as well as inflammation. ATP-dependent K+ channels mediate a part of the observed anti-nociceptive effect.

## Introduction

Pain and inflammation are among the most commonly occurring complications of many diseases. As a defense mechanism, they serve to warn against tissue damage so that the removal of the causing factor is pursued. For relieving pain, non-stroidal anti-inflammatory drugs (NSAIDs) and opioid analgesics are widely used. Inflammation is also controlled using some types of NSAIDs and glucocorticoids (Katzung and Trevor, 2015 ▶). Although opioids are potent analgesics but their side effects including sedation, respiratory suppression, nausea, vomiting, constipation and bladder spasm are bothering and limit their use. Their chronic usage might also cause hypogonadism, immune suppression and addiction (Busse et al., 2018 ▶). NSAIDs might cause cardiovascular complications (e.g. fluid retention, hypertension and edema) and gastrointestinal problems such as stomachache, nausea, vomiting, and gastric ulcers. Exacerbation of asthma as well as kidney damages are the other side effects of NSAID_S_ (Ngo and Addison, 2018 ▶). Glucocorticoids have also several undesirable effects and especially in chronic use they cause osteoporosis, adrenal suppression and ocular complication like cataract and glaucoma (Katzung and Trevor, 2015 ▶).

Considering the above-mentioned side effects, many investigators have focused on herbal remedies as substitutes for chemical agents. 

The Pinaceae or pine family is a large group of plants with more than 220 species in 11 genera, including cedars, firs, hemlocks, larches and spruces in its domain (Ran et al., 2018 ▶). Anti-inflammatory and analgesic properties of some *Pinus* species e.g. Japanese red pine (*Pinus densiflora*), Maritime pine (*Pinus pinaster*), Bosnian pine (*Pinus heldreichii*), Korean pine (*Pinus koraiensis*), Chir pine (*Pinus roxburghii*), and *Pinus sibrica* have been documented (Basholli-Salihu et al., 2017 ▶; Choi, 2007 ▶; Kang et al., 2016 ▶; Kaushik et al., 2012 ▶; Shikov et al., 2008 ▶; Tümen et al., 2018 ▶).

Iranian pine or Tehran pine (*Pinus eldarica*) naturally grows in Iran, Afghanistan, Pakistan and some other countries. In Asia and Russia, it is traditionally used for alleviating asthma, scars and inflammations, allergic rashes and dermatitis (Mamedov et al., 2005 ▶; Zargari, 1996 ▶). 

Different parts of this plant have been examined and their medicinal benefits have been reported. Bark essential oil and extract showed promising cytotoxic and cytoprotective results along with anti-pseudomonal properties (Babaee et al., 2016 ▶; Sadeghi et al., 2016 ▶; Sarvmeili et al., 2016 ▶). Hydroalcoholic extract of needle leaves had anti-depressive effects and increased pentobarbital sleep induction (Bolandghamat et al., 2011 ▶; Forouzanfar et al., 2016 ▶). Seeds of *P. eldarica* reduced blood cholesterol and was effective against hyperlipidemia and arthrosclerosis (Huseini et al., 2015 ▶). 

Study on *P. eldarica* chemical components indicated that the bark contains polyphenols such as taxifolin and catechin, which have anti-inflammatory, anticancer and antioxidant properties (Iravani and Zolfaghari, 2014 ▶; Sarvmeili et al., 2016 ▶). Essential oil obtained from the bark contained alpha-pinene and caryophyllene oxide (Sarvmeili et al., 2016 ▶). Needle leaves were shown to contain tannin, terpenoids and polyphenols (Forouzanfar et al., 2016 ▶). Compounds such as beta-caryophyllene, beta-pinene, alpha-humulene and Junipene were found to be present in its bark and fruits (Afsharypuor and San'aty, 2005 ▶). 

Based on the above information, this study was conducted to evaluate anti-nociceptive and anti-inflammatory effects of hydroalcoholic extract of bark and essential oil of needle leaves of *P. eldarica* in animal models. 

## Materials and Methods

**Chemicals**

Acetic acid and formalin (Merck, Germany), croton oil and carrageenan (Sigma, USA) were used in this research.

**Hydroalcoholic extract preparation**

*P. eldarica* bark (herbarium no. 3318) was fully ground and weighed. The powder was mixed with Methanol-water (90:10) in an Erlenmeyer flask with its top closed by an aluminum foil. Using an electric shaker, materials were mixed for 30 min. The Erlenmeyer flask containing the mixture was stored in darkness for 24 hr. Then, sufficient solvent was again added and mixed as before. The resulting mixture was filtered using the Buchner funnel. In the next step, the same procedure was repeated using methanol-water (1:1). The extract was then fully condensed using a vacuum apparatus. For removing the remaining solvent and further drying the powder, the extract was freeze dried. To prevent any unwanted changes in the final extract and its active components content, it was stored in an airtight container inside a refrigerator (4°C) (Hosseinzadeh et al., 2010 ▶).

**Essential oil preparation**

Fresh *P. eldarica* needle leaves were cut into appropriate size. The Clevenger apparatus was used to prepare the essential oil by hydro-distillation method. The mixture was heated for about 4 hr that is enough time for the leaves to be fully extracted and thus the obtained essential oil reaches a constant amount. The essential oil was carefully obtained and stored in small air-tight containers in a refrigerator (Afsharypuor and San'aty, 2005 ▶). 

**Analysis of the essential oil of** ***P. eldarica*** **leaves using gas chromatography apparatus (GC-MS)**

Using gas chromatography apparatus and following the related standards, 0.1 µl of the essential oil was analyzed. Specifications and circumstances were as follows: the apparatus was connected to Agilent technologies 5975C Mass spectrometer with a 30 m long column with an inner girth of 0.25 mm and covering layer thickness of 0.25 µm. The injector’s temperature was 280°C and column’s temperature was set from 70 to 250°C and 4°C/min gradient. Helium gas was used as the carrier gas with 1.9 ml/min speed and 17.7 pound per square inch pressure. Mass spectrometer was used in electron ionization mode, 70eV voltage, 230°C ionization chamber temperature and 750 microampere ionization current.

**Animal experiments**

**Animals**

Male Swiss mice (25-30 g) and male Wistar rats (180-200 g) were used in this study. Animals were kept in standard conditions of light/dark cycle, humidity and temperature with free access to water and food. Animal experiments were approved by Ethics Committee of Isfahan University of Medical Sciences (IR.MUI.RESEARCH.REC.1398.325). 

**Acetic acid test**

Doses of 100, 200 and 400 mg/kg of the hydroalcoholic extract and 50, 100, and 200 µl/kg of the essential oil and 10 mg/kg indomethacin were administered intraperitoneally (i.p.) to groups of mice (n=6). Hydroalcoholic extract and essential oil were prepared for injection using 0.5% tween 80 in normal saline. One group received only the vehicle without any drug and served as the control group. Thirty minutes later, 10 ml/kg of 1% acetic acid (v/v) was injected i.p. and after 10 min, abdominal contractions were counted for the next following 10 min (Hajhashemi and Dehdashti, 2014 ▶). In two series of animals, to investigate the possible mechanism of anti-nociception, yohimbine (5 mg/kg), ondansetron (0.5 mg/kg), naloxone (5 mg/kg) and glibenclamide (10 mg/kg) were administered (Alonso-Castro et al., 2020 ▶), 30 min prior to hydroalcoholic extract (400 mg/kg) or essential oil (200 µl/kg) and acetic acid test was performed as above.

**Formalin test**

Hydroalcoholic extract (100, 200 and 400 mg/kg), essential oil (50, 100, and 200 µl/kg) and morphine (10 mg/kg) were injected (i.p.) to mice (n=6) and after 30 min, 20 µl formalin (2.5% v/v) was injected into the subcutaneous space of mice paw. The control group received vehicle. Paw licking time was recorded for the first five minutes after the formalin injection as an indicator of acute pain and between the 20^th^ and 40^th^ min of injection, indicating chronic pain (Hajhashemi et al., 2011 ▶). 

**Hot plate test**

Using a hot plate apparatus (Borj Sanat, Tehran, Iran) set at a temperature of 55°C, the control latency for each mouse was measured showing the sensitivity and responding time of each mouse to the heat (control latency). Kicking, jumping or licking the paw was considered as animal reaction. Different doses of hydroalcoholic extract (100, 200, and 400 mg/kg) and essential oil (50, 100, and 200 µl/kg) were administered i.p. to different test groups (n=6). The standard and control groups received morphine (10 mg/kg i.p.) and vehicle, respectively. The process of measuring the reaction time was again repeated every 30 min, 4 times and recorded as the test latency. Cut off time for this test was 20 sec (Hajhashemi and Dehdashti, 2014 ▶). The maximum possible anti-nociceptive effect (MPE%) was calculated as follows:

MPE%= [test latency - control latency]/ [cut-off time - control latency] X 100

**Carrageenan test**

Rats were divided in different groups (n=6) and received different doses of hydroalcoholic extract (100, 200, and 400 mg/kg) and essential oil (50, 100, and 200 µl/kg). The standard and control group received indomethacin (10 mg/kg, i.p.) and vehicle (1 ml/kg, i.p.), respectively. Thirty minutes afterwards, 100 µl of 1% w/v carrageenan was injected into the right paw of the rats. The volume of the right paw was measured for each rat right before and 4 hr after the injection of carrageenan using a plethysmograph (Ugo Basil, Italy). The difference between these two volumes was considered as the amount of edema (Zabihi et al., 2017 ▶). 

**Croton oil-induced ear edema**

Eight groups of male mice (n=6) received hydroalcoholic extract (100, 200, and 400 mg/kg), essential oil (50, 100, and 200 µl/kg), indomethacin (10 mg/kg) or vehicle (1% tween 20 in saline, 10 ml/kg) i.p., 30 min after gentle application of 20 µl croton oil solution (5% v/v in acetone) on the inner surface of right ear. Six hours later, mice were sacrificed and 6-mm (in diameter) disks were cut from the right and left ears. The difference between the weight of disks represented the amount of ear edema (Hajhashemi et al., 2011 ▶).

**Statistical analysis**

Data were analyzed by SPSS (version 21) using one way analysis of variance (ANOVA) followed by Scheffe *post hoc*. The results are expressed as mean±SEM. and differences were considered significant at p-values less than 0.05.

## Results

**Pharmacognosy**

The yield of hydroalcoholic extract and essential oil were 20.4% (w/w) and 0.2% (v/w), respectively. Using the calibration curve equation, the total phenolic content of the extract in terms of gallic acid equivalent was 404.9±7.7 mg/g of the extract powder.

**GC/MS analysis of** ***P. eldarica*** **essential oil**

The essential oil consists of monoterpenes (31%) and sesquiterpenes (69%) and germacrene D (35.72%), β caryophyllene (18.45%) and δ-cadinene (5.53%) were the major constituents. 

**Pharmacology**

**Acetic acid test**

**Figure 1:**
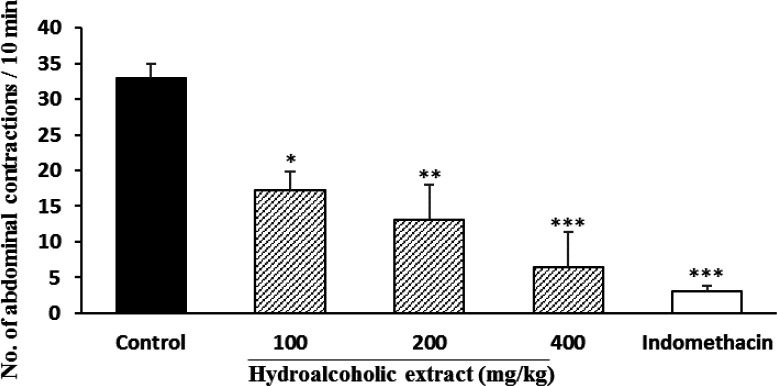
Effect of *P. eldarica* bark hydroalcoholic extract in acetic acid test in mice (n=6). Vehicle (10 ml/kg tween 80 0.5% in saline), hydroalcoholic extract (100, 200 and 400 mg/kg) and indomethacin (10 mg/kg) were injected i.p. 30 min before acetic acid injection (10 ml/kg, i.p.) and after 10 min, the number of abdominal contractions was counted in a 10 min interval. *p<0.05; **p<0.01 and ***p<0.001 compared with the control

**Figure 2:**
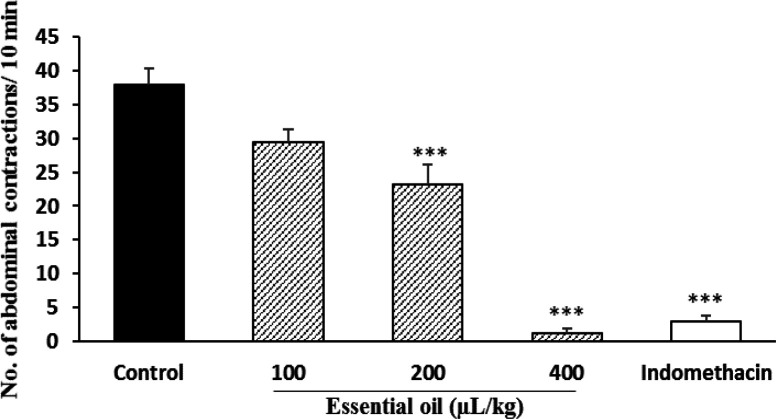
Effect of *P. eldarica* leaves essential oil in acetic acid test in mice (n=6). Vehicle (10 ml/kg tween 80 0.5% in saline), essential oil (50, 100 and 200 µl/kg) and indomethacin (10 mg/kg) were injected i.p. 30 min before acetic acid injection (10 ml/kg, i.p.) and after 10 min, the number of abdominal contractions was counted in a 10 min interval. ***p<0.001 compared with the control

In the acetic acid test, doses of 200 and 400 mg/kg of hydroalcoholic extract reduced the number of abdominal spasms by 60.6 and 80.3% respectively (Figure 1) and essential oil at doses of 100 and 200 µl/kg significantly (p<0.001) reduced the number of abdominal spasms by 39 and 97%, respectively (Figure 2). The percentage of inhibition was 91% for indomethacin. 

**Formalin test**

**Figure 3:**
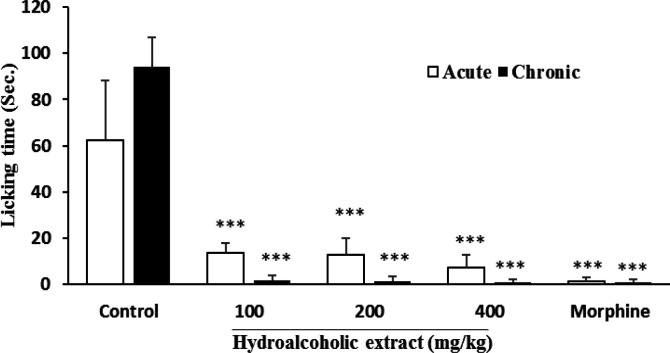
Effect of *P. eldarica* bark hydroalcoholic extract in formalin test in mice (n=6). Vehicle (10 ml/kg tween 80 0.5% in saline), hydroalcoholic extract (100, 200 and 400 mg/kg) and indomethacin (10 mg/kg) were injected i.p. 30 min before subcutaneous injection of formalin (20 µl, 2.5% v/v) into the right hind paw of animals. Licking time was measured 0-5 and 20-40 min after formalin injection and respectively considered as acute and chronic phases. ***p<0.001 compared with the control

**Figure 4:**
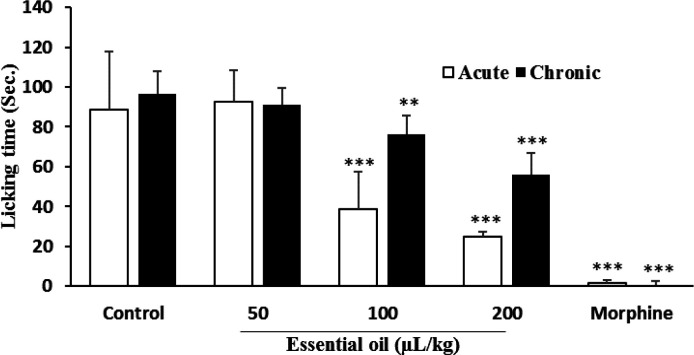
Effect of *P. eldarica* leaves essential oil in formalin test in mice (n=6). Vehicle (10 ml/kg tween 80 0.5% in saline), essential oil (50, 100 and 200 µl/kg) and indomethacin (10 mg/kg) were injected i.p. 30 min before subcutaneous injection of formalin (20 µl, 2.5% v/v) into the right hind paw of animals. Licking time was measured 0-5 and 20-40 min after formalin injection and respectively considered as acute and chronic phases. **p<0.01 and ***p<0.001 compared with the control

Results of formalin test revealed significant (p<0.001) reduction of paw licking time for 100, 200 and 400 mg/kg doses of hydroalcoholic extract with respective inhibition rates of 77.8, 79.2, and 88.3% in acute phase and 98.2, 98.6 and 99.1% in chronic phase. The results for morphine were 97.6% for acute and 99.1% for chronic phase (Figure 3). While a dose of 50 µl/kg of essential oil did not show any significant inhibition of pain behavior, the other tested doses (100 and 200 µl/kg) significantly suppressed pain in both phases. The percentage of inhibition of paw licking for acute and chronic phases of 100 µl/kg essential oil was 56.7 and 21.8% and for the 200 µl/kg dose it was 72.2 and 42% respectively (98.3 and 99.1% respectively for morphine) (Figure 4).

**Hot plate test**

In hot plate test, while morphine (10 mg/kg) demonstrated significant antinociception (p<0.001), neither the hydroalcoholic extract nor the essential oil had antinociceptive activity (Table 1). 

**Table 1:** Effect of *P. eldarica* bark hydroalcoholic extract and leaves essential oil in hot plate test in mice (n=6).

Treatment	Dose	MPE% at different time points			
		30 min	60 min.	90 min.	120 min
Control	-	5	5	3	5
Hydroalcoholic ext.	100 mg/kg	7	7	5	3
	200 mg/kg	11	7	5	3
	400 mg/kg	9	5	5	5
Essential oil	50 µl/kg	7	7	6	6
	100 µl/kg	7	9	5	5
	200 µl/kg	5	9	7	5
Morphine	10 mg/kg	61***	95***	45***	31***

Different doses of the hydroalcoholic extract (100, 200, and 400 mg/kg) and essential oil (50, 100, and 200 µl/kg) were administered i.p. to different test groups. The standard and control groups received morphine (10 mg/kg i.p.) and vehicle, respectively. The reaction time on hot plate was measured just before treatments (control latency) and every 30 min, 4 times (test latency). Cut off time for this test was 20 sec. The maximum possible anti-nociceptive effect (MPE%) at each time point was calculated as follows: MPE%= [test latency - control latency]/ [cut-off time - control latency] X 100. ***p<0.001 compared with the control group.

**Carrageenan test**

In carrageenan test, all three tested doses of hydroalcoholic extract and the maximum tested dose of the essential oil (200 µl/kg) significantly (p<0.05) decreased carrageenan-induced paw edema (Table 2).

**Croton oil test**

As it is seen in Table 3, in croton test, the extract at doses of 100, 200 and 400 mg/kg 

and the essential oil at doses of 100 and 200 µl/kg were effective (p<0.05).

**Effect of antagonists on antinociceptive effect**

**Figure 5:**
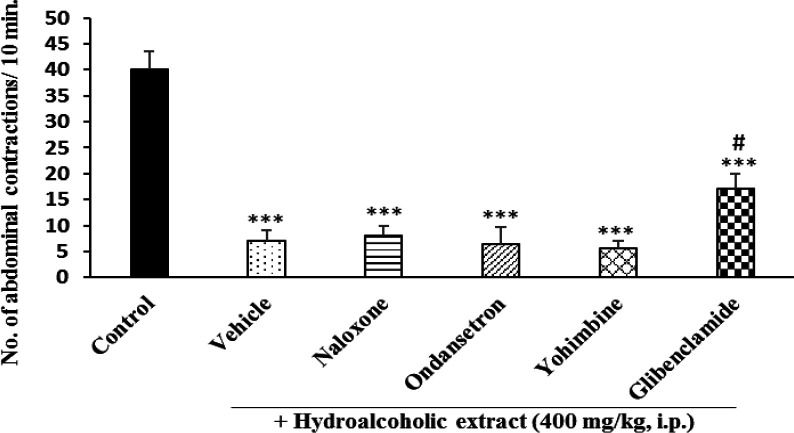
Effect of different antagonists on antinociceptive effect of *P. eldarica* hydroalcoholic extract. Four groups of mice (n=6) were pretreated with naloxone (5 mg/kg), ondansetron (0.5 mg/kg), yohimbine (5 mg/kg) and glibenclamide (10 mg/kg) and one group received vehicle (10 ml/kg tween 80 0.5% in saline). Thirty minutes later, the hydroalcoholic extract (400 mg/kg, i.p.) was administered to all animals and after 30 min, acetic acid (10 ml/kg, i.p.) was injected. The control group received only acetic acid. The number of abdominal contractions was counted in a 10-min period started 10 min after acetic acid injection. ***p<0.001 compared with the control. #p<0.05 compared with the hydroalcoholic extract group

**Figure 6:**
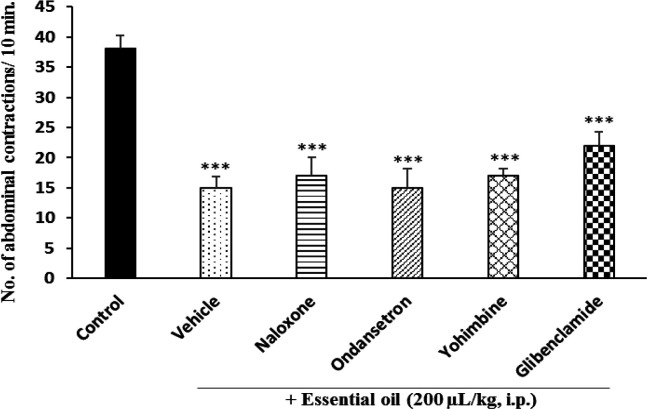
Effect of different antagonists on antinociceptive effect of *P. eldarica* essential oil. Four groups of mice (n=6) were pretreated with naloxone (5 mg/kg), ondansetron (0.5 mg/kg), yohimbine (5 mg/kg) and glibenclamide (10 mg/kg) and one group received vehicle (10 ml/kg tween 80 0.5% in saline). Thirty minutes later, the essential oil (200 µl/kg, i.p.) was administered to all animals and after 30 min, acetic acid (10 ml/kg, i.p.) was injected. The control group received only acetic acid. The number of abdominal contractions was counted in a 10-min period started 10 min after acetic acid injection. ***p<0.001 compared with the control

While pretreatment of animals with naloxone, ondansetron and yohimbine could not reverse the antinociceptive effect of *P. eldarica* hydroalcoholic extract, glibenclamide partially antagonized this effect (Figure 5). In case of the essential oil, none of the antagonists was effective in reversing the antinociceptive effect (Figure 6).

**Table 2:** Effect of *P. eldarica *bark hydroalcoholic extract and leaves essential oil in carrageenan test in rats (n=6)

Treatment	Dose	Mean of paw edema (ml)	% inhibition
Control	-	0.73	-
Hydroalcoholic ext.	100 mg/kg	0.42	42.5*
	200 mg/kg	0.43	41.1*
	400 mg/kg	0.36	50.7*
Essential oil	50 µl/kg	0.61	16.4
	100 µl/kg	0.59	19.2
	200 µl/kg	0.38	47.9*
Indomethacin	10 mg/kg	0.14	80.8***

Different doses of hydroalcoholic extract and essential oil were administered (i.p.) 30 min prior to subcutaneous injection of carrageenan (100 µl, 1% w/v) into the right hind paw and paw volume was measured at hour 0 and 4 and the volume difference was taken as paw edema. The control and reference groups received vehicle (1 ml/kg) and indomethacin (10 mg/kg) respectively. % inhibition= [(mean of paw edema in control group- mean of paw edema in test group)]/ mean of paw edema in the control group X 100. *p<0.05 and ***p<0.001 in comparison with the control group.

**Table 3:** Effect of *P. eldarica* bark hydroalcoholic extract and leaves essential oil in croton test in mice (n=6)

Treatment	Dose	Mean of ear edema (mg)	% inhibition
Control	-	15.78	-
Hydroalcoholic ext.	100 mg/kg	8.85	43.9*
	200 mg/kg	8.15	48.4*
	400 mg/kg	7.62	51.7*
Essential oil	50 µl/kg	13.33	15.5
	100 µl/kg	11.07	30.0*
	200 µl/kg	11.08	29.8*
Indomethacin	10 mg/kg	2.7	82.9***

Vehicle, different doses of the hydroalcoholic extract or the essential oil or indomethacin were administered (i.p.) 30 min prior to topical application of croton oil (5% in acetone, 20 µl) on the right ear. Six hours later, the animals were sacrificed and 6-mm disks were taken from both ears and weighed. The weight difference was an index of edema. % inhibition= [(mean of ear edema in control group- mean of ear edema in test group)]/ mean of ear edema in the control group X 100. *p<0.05 and ***p<0.001 in comparison with the control group.

## Discussion

The results of the present study clearly showed that hydroalcoholic extract of bark and essential oil of needle leaves of *P. eldarica* are capable of reducing pain and inflammation to a significant degree.

Previously, analgesic and anti-inflammatory effects were reported for some other species of *Pinus* including *Pinus roxburghii*, *Pinus sibirica*, *Pinus densiflora,* *Pinus pinaster,* *Pinus* heldreichii and *Pinus koraiensis, *and *Pinus rigida* (Basholli-Salihu et al., 2017 ▶; Choi, 2007 ▶; Jang et al., 2008 ▶; Jin et al., 2017 ▶; Kang et al., 2016 ▶; Kaushik et al., 2012 ▶; Shikov et al., 2008 ▶; Tümen et al., 2018 ▶). To the best of our knowledge, this is the first report on antinociceptive and anti-inflammatory effects of *P.* *eldarica*.

To evaluate the effectiveness of this plant several tests were conducted in animal 

models each inducing pain and edema in different ways.

The result of acetic acid test was very promising as treatments provided adequate suppression of abdominal pain. Acetic acid writhing test has been widely used as an animal model of inflammatory pain and shows similarities to visceral pain in human. In this test, following i.p. injection of acetic acid, several pro-inflammatory mediators and the products of arachidonic acid pathway, especially prostaglandin E_2_ (PGE_2_)_, _stimulate peripheral nociceptors and induce abdominal spasms (Zulfiker et al., 2010 ▶). 

 In formalin test, both the hydroalcoholic and essential oil of *P. eldarica *could suppress pain behavior. In this test, in the acute phase, which starts just after formalin injection, nociceptors are stimulated and they relay pain signal via C fibers and the chronic phase (second phase) is mostly due to paw inflammation (Hajhashemi and Klooshani, 2013 ▶). Our results obtained from formalin test indicated that the pain killing activity of *P. eldarica* on chronic phase was more pronounced, and these results were confirmed by carrageenan and croton oil tests that assessed anti-inflammatory effects.

It has been shown that flavonoids inhibit biosynthesis of prostaglandins. They are also able to suppress neutrophils degranulation and decrease the release of arachidonic acid which is the precursor of prostaglandins (Agrawal, 2011 ▶). Therefore, flavonoids of the hydroalcoholic extract of the plant might contribute to its anti-inflammatory activity.

 It has been reported that *P. eldarica* extract has antioxidant activity (Asgharzadeh et al., 2016 ▶). Also, the crucial role of flavonoids in reducing oxidative stress-mediated pain and inflammation is well- documented (Vazhappilly et al., 2019 ▶). Based on these studies, it is concluded that antioxidant effect of *P. eldarica* extract might also contribute to the effects observed in our study. 

Several neurotransmitters including endogenous opioids, nitric oxide, monoamines such as serotonin and norepinephrine as well as substance P, are known as the major targets for pain and inflammation (McCurdy and Scully, 2005 ▶). To figure out if the extract or the essential oil of *P. eldarica* affects any of the common receptors involved in the analgesic response, different drugs were administered prior to the extract or the essential oil, and the acetic acid test procedure was followed. Naloxone, ondansetron, yohimbine as antagonists of opioid, 5-HT_3_ serotonin and α_2_ adrenergic receptors and glibenclamide as an ATP-dependent K^+^-channel blocking agent were injected intraperitonially prior to administration of the extract or essential oil and except glibenclamide which partially reversed the antinociceptive effect of the hydroalcoholic extract, other tested antagonists could not exert any significant changes in analgesia, indicating that these receptors are not involved in the pain killing effect. It has been documented that K_ATP_ channels are activated via nitric oxide (NO)/cGMP/cGMP-dependent protein kinase pathway. Activation of these channels is associated with hyperpolarization of the neuronal cells that are involved in pain signaling (Chai and Lin, 2010 ▶). In our study, partial reversal of anti-nociceptive effect of hydroalcoholic extract of *P. eldarica* by glibenclamide might indicate the partial involvement of NO/cGMP/cGMP protein kinase pathway. Further studies are needed to present a conclusive and definite mechanism of action.

Since several neurotransmitters and pathways contribute to pain signaling, further studies are suggested to find out the exact mechanism of pain killing effect of the plant extract or the essential oil.

Generally concluding, the essential oil of needle leaves and the hydroalcoholic extract of bark of *P. eldarica *were shown to be effective in alleviating acute and chronic pain in various tests and therefore they are promising substitute for conventional chemical drugs currently being used. Future pharmaceutical and clinical studies are needed to make possible the formulation and usage of this plant in humans. 
